# The Influence Exerted by Time Frames on Consumers’ Willingness to Buy Nearly Expired Food

**DOI:** 10.3389/fpsyg.2021.790727

**Published:** 2021-12-20

**Authors:** Mingrui Song, Yijun Zhao, Xianguo Li, Lu Meng

**Affiliations:** Business School, Renmin University of China, Beijing, China

**Keywords:** time frames, nearly expired food, time perception, delay, date

## Abstract

With the development of Internet e-commerce channels, online shopping platforms have become the main channel for consumers to buy nearly expired food. Date labels, as one of the main external clues, play a decisive role in nearly expired food purchasing. Therefore, based on attention-related theory, this study attempts to explore the influence exerted by different time frames on consumers’ willingness to buy and its mechanism. The results show that compared with the date, consumers have a higher willingness to buy nearly expired food when the expiration time is framed by delay. More specifically, compared to the date, the delay causes the individual to have a longer time perception, thus more preference for nearly expired food. Meanwhile, the mediating effect of time perception is moderated by food type. The conclusion of this research is helpful to expand the theoretical framework of time frames and related fields on nearly expired food, as well as provide practical guidance for marketers to effectively promote nearly expired food.

## Introduction

There are many foods on sale with “close to expiration date” as the key-words on the existing e-commerce platforms, such as Taobao, Jingdong, and Suning Tesco.^1^ Data shows that when searching with the keywords “close to expiration date,” the total number of foods is 40,615 on Taobao, 8,786 on Jingdong, and 2,433 on Suning Tesco. From the top five of the volume-ranked nearly expired food sales, seemingly, it appears that consumers prefer to buy packaged snacks.^2^ It can be seen that multiple channels of the online platform can meet the needs of consumers to buy nearly expired food. Most of the reasons why consumers purchase nearly expired food are that a considerable number of people still have high sensitivity to commodity prices, and consumption degradation characterized by “low price, de-branding, good quality, basic functions” has become a phenomenon that cannot be ignored. Therefore, the main driving force for consumers to buy nearly expired food in 2020 is to buy the goods they need at low prices, which means the high preference of price-judgement. On the other hand, studies have shown that most food waste, at the retail stage, is associated with the expiration date of the food (Garrone et al., 2014), namely, “beyond the shelf-life” is one of the important reasons for consumers to discard and waste food (Samotyja and Sielicka-Różyńska, 2021). Therefore, the hunger problem caused by the food crisis phenomenon is also another important incentive for consumers to purchase nearly expired food online. Although the sale of nearly expired food online has a certain price advantage, e.g., the rental fee of an online store is at a lower price or even totally free, nearly expired food does still have a certain production cost. If the price is too low, it will trigger the damage of the brand’s image. Therefore, compared with blindly reducing the price of nearly expired food to obtain consumers’ purchases, marketers need to consider changing other marketing clues of nearly expired food to enhance consumers’ willingness to buy it.

Previous studies on nearly expired food mainly focused on pricing (Theotokis et al., 2012; Aschemann-Witzel, 2018), date label (Collart and Interis, 2018; Wilson et al., 2018; Samotyja and Sielicka-Różyńska, 2021), consumer personal characteristics (de Hooge et al., 2017; Konuk, 2018), and other contextual factors as social environment (do Carmo Stangherlin et al., 2020). Compared with the elements, personal characteristics for example, that are difficult for marketers to change, the price and the date label of nearly expired food can be changed by marketers, but the manipulation of the date label of nearly expired food is more convenient and concise than the cost loss caused by the situation factor and the price. In addition, compared with other food, nearly expired food has the characteristic of having a shorter edible time window, so the date label information is the most important product clue in the process of purchasing. However, the existing research on the date label of nearly expired food mainly focuses on the differences between date labels (Wilson et al., 2017, 2018; Li et al., 2020; Samotyja and Sielicka-Różyńska, 2021) and consumer knowledge of shelf-life (Manzocco et al., 2016; Melbye et al., 2016; Collart and Interis, 2018). Few studies have focused on the influence exerted by the time frames of expiration time on the purchase of nearly expired food. Previous studies have shown that although there is an objective and unified measure of time, different time frames will lead to consumers having different perceptions. Specifically, the accuracy of time (Chandran and Menon, 2004; Charles Zhang and Schwarz, 2012) and unit (Lembregts and Pandelaere, 2013) have an effect on consumers’ psychology and behavior. In the actual consumption situation, nearly expired food sold on online platforms such as HAO SHIQI,^3^ the information of expiration time is marked as “expired on day/month/year” or “expired in XX (days),” that is framed as the date or the delay, respectively. This research attempts to explore whether the expiration time formed by the time frames of date and delay have different effects on psychology, and which kind of time frame can increase consumers’ willingness to buy nearly expired food. The contribution of this research can be concluded into two points. Firstly, the research hopes to extend the time frames and the relevant theoretical framework of consumption to nearly expired food. Secondly, to improve the sales volume of nearly expired food in analyzing consumers’ psychology and purchasing behavior, and provide theoretical support and guidance suggestions for the sales and strategies of nearly expired food.

## Theoretical Framework

### Nearly Expired Food

Nearly expired food refers to products that deviate from normal or optimal products on the basis of their date labelling (e.g., close to expiration date; de Hooge et al., 2017). The existing research on nearly expired food mainly focuses on pricing (Theotokis et al., 2012; Aschemann-Witzel, 2018) and date labels (Collart and Interis, 2018; Wilson et al., 2018; Samotyja and Sielicka-Różyńska, 2021). In the research of pricing, research mainly focuses on the impact of expiration date-based pricing (EDBP) on consumers’ decision-making, because EDBP is one of the most common pricing strategies for marketers to sell nearly expired food. For example, Theotokis et al. (2012) find that EDBP will have a negative impact exerted by consumers on the perception of the quality of nearly expired food, because this pricing strategy violates the individual’s psychology; On the contrary, for consumers familiar with such pricing, EDBP has no effect on the brand quality and image of nearly expired food. Aschemann-Witzel (2018) mainly explores which situation can EDBP increase consumers’ purchases. The research results show that compared with promoting EDBP to avoid food waste and help save their budget, improving consumers’ familiarity with EDBP can increase consumers’ purchases.

The date labels include “BEST BY,” “USE BY,” “SELL BY,” “FRESH BY” and “EXPIRATION ON.” In the research of date labels of nearly expired food, some studies compared the difference between the date labels. For example, research shows that “USE BY” is the food safety indicator with the least ambiguous and “SELL BY” is the food safety indicator with the most ambiguous. Therefore, consumers have the highest willingness to waste food that exceeds “USE BY,” and have the lowest willingness to waste food that exceeds “SELL BY” (Wilson et al., 2017). Some researchers also pointed out that consumers are more likely to consume food if it exceeds the “BEST BY” rather than the “USE BY.” The reason is that consumers intuitively believe that the “BEST BY” represents quality, while the “USE BY” represents safety (Wilson et al., 2018). Other studies have shown that consumers are more likely to use appearance as a selection criterion when the date label is the “BEST BY,” while consumers are more likely to use date as a selection criterion when the date label is the “USE BY” (Samotyja and Sielicka-Różyńska, 2021). In addition, Li et al. (2020) found that no matter what type of date information, once it appears, consumers will lower the evaluation of the food that exceeds that date. Therefore, the author proposes to set a date label that represents safety rather than taste to extend the time of the sale of the food, thereby increasing consumer purchases. At the same time, some studies have also explored consumers’ knowledge of date labels. For example, when consumers have less knowledge, they are more inclined to waste food past the ‘best before’ (Manzocco et al., 2016; Melbye et al., 2016). Collart and Interis (2018) find that increasing consumers’ knowledge of shelf-life labels and environmental awareness can promote consumers’ preference for food beyond “BEST BY.” Besides the studies above, a few scholars have paid attention to the influence of consumer personal characteristics (de Hooge et al., 2017; Konuk, 2018) and contextual factors (do Carmo Stangherlin et al., 2020) on nearly expired food purchasing.

To sum up, the main driving factors for the purchase of nearly expired food are price and date label (Theotokis et al., 2012; Aschemann-Witzel, 2018; Collart and Interis, 2018; Wilson et al., 2018; Samotyja and Sielicka-Różyńska, 2021). Compared with the research conclusions that the pricing strategy is more consistent, the existing research on how different frames of the date label affect consumers’ decision-making of nearly expired food is not deep enough. Research has mainly explored the effect of different date labels and the consumer knowledge, while not paying attention to the influence of time frames about expiration time on consumer psychology and behavior. Therefore, it is a new research perspective in the field of nearly expired food to explore consumers’ willingness to buy nearly expired food from different time frames (date and delay).

### Time Frames

Most studies have found that consumers have different cognitive effects on the same length of time in different time frames. Formerly, the representation of time frames has mainly focused on how the time precision and unit affect the change of consumer behavior. As shown by Chandran and Menon (2004), when health hazard data are represented on “a day,” rather than on “a year,” the individual’s perceived risk is made more specific, thereby increasing self-perception of risk. Charles Zhang and Schwarz (2012) found that when individuals estimate future budgets, lower time accuracy (for example, 7years, compared to 84months) will make the individual perceive greater difficulty and lower self-confidence in the estimation, and individuals will continue to adjust the estimated value of the future budget. As a result, the estimated value is closer to the true value. Besides, the unit of time will also affect consumers’ attitude and evaluation on products. For example, study has shown that when the service life and warranty period of a product is presented in default units (compared to uncommon units), consumers come to the realization that the product is more attractive and have a higher willingness to buy it (Lembregts and Pandelaere, 2013).

What is more relevant to this study is that in the daily consumption of the nearly expired food, enterprise often adopted two kinds of time frames propagating the information to describe the expiration time by date (eg: day/month/year) or delay [eg: XX (days)]. In this research, the date is a date-based time interval frame while the delay is a time interval frame based on the number of days. Existing studies have not revealed how the time frames affect consumers’ consumption decisions. Accordingly, this study carries out in-depth exploration from this perspective. Formerly, most research on date and delay mainly focused on the field of intertemporal decision-making (Read et al., 2005; Leboeuf, 2006; Malkoc et al., 2010; Munichor and Leboeuf, 2018). For example, some scholars have pointed out that when a future transaction is described in terms of delay rather than date, individuals will have a higher discount rate on future transactions (Read et al., 2005). Some scholars found the influence of time frames on individual discount, namely, the time described by the delay with the increase of time range, the lower people discount on future results, while the frame of the date does not produce a similar phenomenon (Zauberman et al., 2009; Malkoc et al., 2010). Dshemuchadse et al. (2013) explored the mechanism by observing the subjects’ mouse movement in intertemporal decision-making, and found that the time frames will affect the individual’s weighting of time and value. Specifically, individuals in the date have a higher weight on value, so they will be more likely to choose the larger-delayed reward than the smaller-immediate reward. Besides, the research in the intertemporal field, some scholars have explored the mechanism of influence of date and delay on future planning (LeBoeuf and Shafir, 2009), goal decision-making (Munichor and Leboeuf, 2018). For example, when the delay is used to estimate the time point at which a future event occurs, compared to the date, individuals underestimate the time at which a future event occurs due to the anchoring adjustment effect (LeBoeuf and Shafir, 2009). Individuals are more likely to pursue goals when the delay is used to describe the time interval to complete the goal rather than the date (Munichor and Leboeuf, 2018). Although scholars have carried out in-depth research on the time frames in the fields of intertemporal decision-making and goal, the existing research rarely explores how their application affects consumers’ preferences and choices. Under the situation of nearly expired food consumption, date and delay are not only the time frames commonly used in promoting food consumption, but the time clues that consumers mainly pay attention to in the decision-making of nearly expired food consumption as well (Tsiros and Heilman, 2005). Therefore, this research intends to explore how the time frames of date and delay affect the individual’s intention to purchase nearly expired food.

## Research Assumptions

### Time Frames, Time Perception, and Willingness to Buy Nearly Expired Food

Previous studies have shown that when time is described as the interval between two dates (the distance between “now” and the “future”), it will appear relatively abstract, because the date itself appears to be a relatively abstract point in time (Leboeuf, 2006), It is difficult for individuals to process abstract date information into specific time length (like “1st January to 3rd February” into 33days), due to individuals preferring information with lower cognitive needs (Kool et al., 2010). In this case, they will avoid processing time lengths, which leads to insufficient attention to the length of the specific time interval in date (LeBoeuf and Shafir, 2009). In contrast, the delay presents a more specific time interval, which makes it easier for individuals to notice the specific interval of time (Leboeuf, 2006), because when the same interval is described by the range of days, the amount of time is highlighted. Therefore, compared with the date, consumers will more viscerally consider the time that will be spent during the interval. Consumers will pay more attention to the specific time interval length when the time interval is framed as a delay instead of a date. Based on the attentional gate model, when individuals allocate more attention resources to time length information, they will perceive that the same time interval is longer (Zakay, 2005). Specifically, the attention gate theory believes that when an individual invests a large amount of attention resources for timekeeping, the attention gate will open, and more pulses will flow from the pacemaker through the attention gate to the cognitive counter (Zakay and Block, 1997), and the greater the number of cognitive counters flowing to, the longer the estimated time interval of the individual. A large number of previous studies have also confirmed that the increased time-awareness will lead to overestimation of time intervals (Klapproth, 2007). When the individual’s attention is focused on the length of time, it will make the estimate of the time interval longer (Klapproth, 2012). It can be seen that since the individual’s attention will be more focused on the length of remaining shelf-life in the delay, based on the attention gate theory, this research believes that individuals will perceive the remaining shelf-life to be longer.

Based on the research of Dörnyei and Gyulavári (2015), the main motivation for consumers to search for label information is to avoid health-related risks. Furthermore, when consumers think that the product is riskier, the date label is important information for consumers to decide whether to accept the product (Yeung and Morris, 2001). Consumers will pay attention to the date label when buying nearly expired food and use it as a criterion for judging the risk. More importantly, when the remaining shelf-life of the food keep decreasing, the consumer’s evaluation of the various dimensions of the food will also decrease. For example, when the food is close to the expiration date, consumers’ perception of its safety is reduced (Newsome et al., 2014), which increases perception of the risk of buying and the frequency of checking food expiration date information (Tsiros and Heilman, 2005). In addition, the shorter the remaining shelf-life of the food, the lower evaluation of its appearance and other sensory attributes, and the easier it is to refuse to buy the food (Samotyja and Sielicka-Różyńska, 2021). It can be seen that in nearly expired food purchasing, when the remaining shelf-life is gradually reduced, it will trigger consumers’ risk perception, thereby reducing consumers’ purchasing. In summary, this research believes that in nearly expired food consumption, consumers will allocate more attention resources to the length of the remaining shelf-life when it is framed as a delay instead of a date, which leads to a longer time perception of the remaining shelf-life. When consumers perceive the remaining shelf-life to be longer, the consumer’s risk perception is alleviated, thereby enhancing consumers’ willingness to buy nearly expired food. Accordingly, this research proposes the following hypotheses:

H1: Compared with the date, consumers have higher willingness to buy nearly expired food when the expiration time is framed by delay.

H2: Time perception plays a mediating role in the influence of time frames on consumers’ willingness to buy nearly expired food. Specifically, compared with the date, consumers have a longer time perception of the remaining shelf-life when the expiration time is framed by delay, thus having a higher willingness to buy nearly expired food.

### Moderation Effect of Food Type

Food can be divided into healthy and unhealthy food (Nagpal et al., 2015). Consumption of healthy food is often associated with a ‘health’ goal, while unhealthy food is often associated with a ‘pleasure’ goal (Raghunathan et al., 2006; Wansink and Chandon, 2006). Healthy food provides more utilitarian attributes to consumers, while unhealthy food provides more hedonic attributes to consumers (Nagpal et al., 2015; Biswas et al., 2017). Due to the different benefits brought to consumers by healthy and unhealthy food, consumers pay more attention to utilitarian attributes when buying healthy food, while they pay more attention to hedonic attributes when buying unhealthy food. Further, shelf-life information is a way to describe the quality, health, and other utilitarian attributes of food (Newsome et al., 2014). Therefore, compared with healthy food, consumers pay less attention to the utilitarian attribute of shelf-life information when buying unhealthy food. When it comes to unhealthy food, consumers pay less attention to the utilitarian attribute of the food, and are less sensitive to the expiration time information, so they will not devote too much attention to different time frames, which causes individuals to have no significant difference between the length of the remaining shelf-life described by the time frames, so that the difference in the expression of the time frames will not cause consumers to buy nearly expired food. On the contrary, when it comes to healthy food, consumers will pay attention to the utilitarian attributes of the food, thus it is more sensitive to the expiration time information. At this time, consumers pay attention to the length of the expiration time described in different time frames differently, which affects their perceptions of the length of remaining shelf-life, thus promoting consumers’ purchase of nearly expired food. Based on this, this research proposes the following hypotheses:

H3: The food type plays a moderating role in the influence of the time frames on consumers’ willingness to buy nearly expired food.

H3a: When the food type is healthy, compared with the date, consumers have a longer time perception of the remaining shelf-life when expiration time is framed by delay, thus having higher willingness to buy nearly expired food.

H3b: When the food type is unhealthy, there is no significant difference between the impact of the time frames on time perception, thus there is no significant difference in consumers’ nearly expired food purchase intentions.

**Figure 1:**
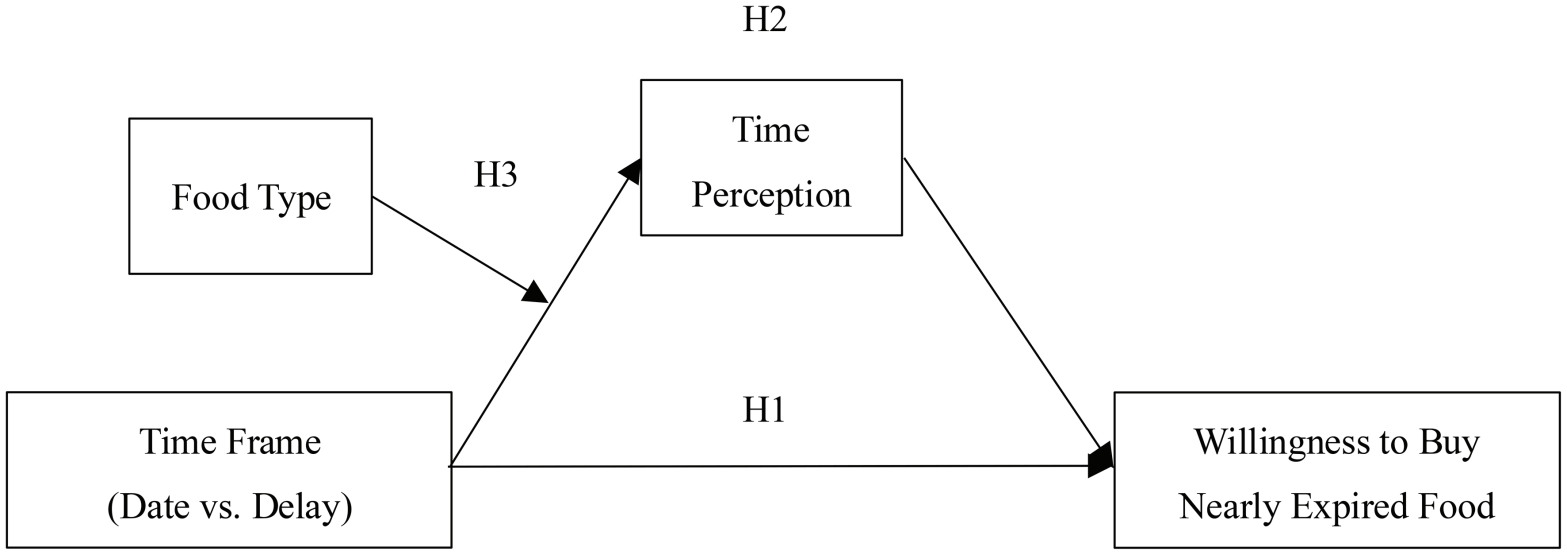
Hypothetical model.

To sum up, the hypothetical framework model of this study is shown in Figure 1, which will be tested by three experiments below.

## Experiment 1

The purpose of Experiment 1 was to verify the different effects of time frames on consumers’ willingness to buy nearly expired food. In particular, compared with date, consumers have higher willingness to buy nearly expired food when the expiration time is framed by delay.

### Participants

Ninety Nine participants (58.6% female; *M*_age_=28.6, *SD*_age_=5.54) were recruited in the experiment. Study 1 employed a 2 (time frames: date vs. delay) between-subjects design.

### Procedure

Firstly, the participants were randomly assigned to two experimental groups and told that the main purpose of the experiment was to test consumers’ preference for nearly expired food. After that, the participants would browse the pictures and related information of the nearly expired food online, and the participants in different groups would see different time information of the nearly expired food. Specifically, product-related information includes product name, specification, shelf-life, and note of expiration time. The stimulus was bread. In order to exclude the influence of brand familiarity, the virtual food brand “YOUPIN” was selected in this study. As for the setting of nearly expired food, as there is no national standard for the definition of nearly expired food, and the definition standard of each province and city is mainly based on the “Zone System of Nearly Expired Food Sales” issued by Beijing.^4^ According to different food shelf-life, the degree of nearly expired food is set in the range of meeting the standard of nearly expired food. In Experiment 1, since the shelf-life of bread was generally in the range of 30days to less than 90days, the degree of expiration was set to 10days. Reference for the manipulation of the time frame for food expiration time is made to the study by Munichor and Leboeuf (2018). The date is expressed as “21st June, 2021” and the delay is expressed as “in the remaining 10days” (see Supplementary Figure A for specific stimuli). Then, the participants answered the purchase intention item about “when you see the product, how likely would you buy it?”(1=very unlikely, 9=very likely; Berry et al., 2017). Finally, the subjects filled in demographic information and were told the true purpose.

### Results

#### Purchase Intention

Taking consumer purchase intention as the dependent variable and the time frames as the independent variable for ANOVA, the results show that consumers in the delay have higher purchase intention to the nearly expired food than that of the date [*M*_delay_=5.59, *SD*_delay_=2.58, *M*_date_=4.54, *SD*_date_=2.55; *F* (1,97)=4.16, *p*=0.044].

### Discussion

Experiment 1 preliminarily tested the influence of time frames on consumers’ purchase intention to the nearly expired food. The results show that compared with the date, consumers in the delay have higher purchase intention to the nearly expired food. In Experiment 2, we will change the stimulus, further verify the main effect, and explore the intermediary mechanism. At the same time, eliminate variables that may cause interference in the experiment.

## Experiment 2

Experiment 2 has three main purposes: firstly, the results of study 1 were repeated by changing the stimulus, that is, to verify the impact of time frames on consumers’ purchase intention to the nearly expired food; secondly, to explore the mediating role of time perception in the influence of time frames on consumers’ purchase intention to the nearly expired food. Specifically, compared with the date, consumers have longer time perception of the remaining shelf-life when expiration time is framed by delay, thus having higher willingness to buy nearly expired food; Thirdly, previous studies have shown that the expression of the delay is more direct and concise than the date, which may lead to higher cognitive fluency and higher willingness to buy nearly expired food. Therefore, this study added a measurement of cognitive fluency to exclude it as a possible underlying mechanism.

### Participants

Two hundred and eighty subjects were recruited through the Marketing Lab platform, of which 259 passed the attention test (64.9% female; *M*_age_=25.5, *SD*_age_=8.15). The experiment uses a single factor (time frames: date vs. delay) to design between-group.

### Procedure

The procedure for Experiment 2 was similar to that for Experiment 1. Firstly, the subjects were told that the study mainly tested the consumers’ preference for the nearly expired food, and then the subjects were given online browsing of the pictures and related information of the nearly expired food, including the product name, specification, production date, shelf-life and expiration time. The stimulus was changed to biscuits with long shelf-life. At the same time, excluding the influence of brand familiarity, the study selected foreign food brand, “LOTUS.” The manipulation of the time frames for the food expiration time was the same as in Experiment 1. As for the degree nearing its expiration date, according to the relevant regulations, the degree of biscuits was set within 45days because the shelf-life of biscuits was usually 1year or more. Among them, the date group was expressed as “19th September, 2021”, and the delay group was expressed as “in the remaining 42days” (see Supplementary Figure B for specific experimental stimuli). After that, the participants were asked to evaluate the purchase intention (Berry et al., 2017), time perception of remaining shelf-life and cognitive fluency. The measurement of time perception, whose entitle was “how long do you think it is before the expiration date for the nearly expired food in the figure above?”(1=very short, 9=very long; Leboeuf, 2006). At the same time, measurement of cognitive fluency, whose entitle was “Based on the product information, you can easily know how long the product will expire” and “Based on the product information, you can quickly know how long the product will expire” (*α*=0.924; 1=very disagree, 9=very agree; Lee and Aaker, 2004). Finally, the subjects filled in demographic information and were told the true purpose.

### Results

#### Time Perception

There were significant differences in the time perception of the remaining shelf-life of nearly expired food under different time frames (date vs. delay). Compared with the date, participants’ perception of remaining shelf-life time in the delay was significant longer [*M*_delay_=4.57, *SD*_delay_=1.91, *M*_date_=4.06, *SD*_date_=1.97; *F* (1,257)=4.50, *p*=0.035].

#### Purchase Intention

Taking the consumer’s purchase intention as variance, time frames as independent to analyze, results showed that compared with date, the participants in the delay have higher purchase intention of nearly expired food [*M*_delay_=5.68, *SD*_delay_=2.16, *M*_date_=5.09, *SD*_date_=2.13; *F* (1,257)=4.83, *p*=0.029].

#### Mediation Analysis of Time Perception

**Figure 2:**
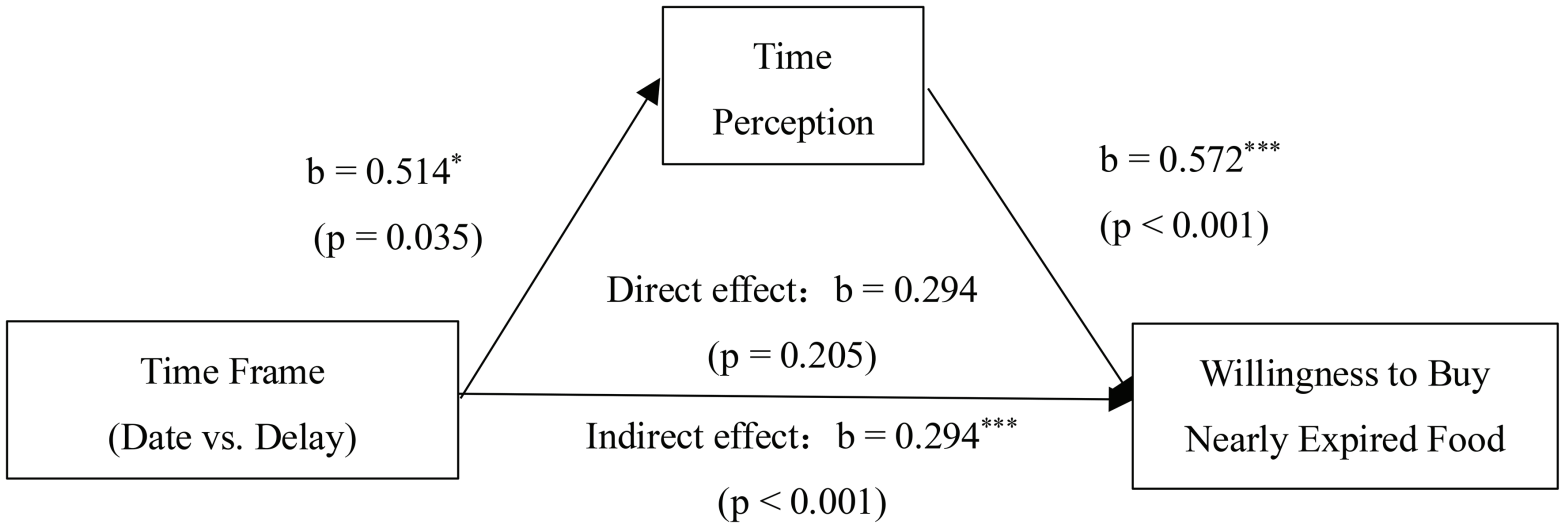
Mediation effect of time perception. ^*^p<0.05; ^**^p<0.01; ^***^p<0.001.

In order to verify the mediating role of time perception in the influence of different time frames on the purchase intention of nearly expired food, this study conducted a test by Bootstrap (Hayes, 2013). When the sample size was 5,000, under the 95% confidence interval, the direct effect result of mediation test included 0 and was not significant (LLCI=−0.1619, ULCI=0.7494), while the indirect effect did not include 0 and was significant (LLCI=0.0288,ULCI=0.5890), indicating that the mediation effect of time perception was significant. The mediation analysis results are shown in Figure 2.

#### Cognitive Fluency

The research results showed that the subjects have no significant difference in cognitive fluency between the date and the delay [*M*_delay_=7.20, *SD*_delay_=1.54, *M*_date_=7.35, *SD*_date_=1.43; *F* (1,257)=0.68, *p*=0.411], which means that the influence of cognitive fluency is excluded.

### Discussion

In experiment 1, bread with a short shelf life was selected as the stimulus. To increase the validity of the results, experiment 2 changed the stimulus and chose biscuits with a longer shelf life. Further, experiment 2 revalidated the main effect and excluded cognitive fluency as an interfering mechanism. The results show that compared with the date, consumers in the delay have a longer perception of the remaining shelf-life of nearly expired food, and thus have a higher willingness to buy. Experiment 3 further verifies the regulating effect of food types, and explores how the time frame affects consumers’ time perception and purchase intention when consumers face different types of nearly expired foods.

## Experiment 3

The main purpose of Experiment 3 is to prove that food type has a moderating effect on the mediating effect of time perception. In particular, when the food type is healthy, compared with the date, the delay can increase consumers’ perception of the time of the remaining shelf-life, thereby increasing consumers’ willingness to buy nearly expired food. When the food type is unhealthy, there is no significant difference between the impact of the time frames on time perception, so there is no significant difference in consumers’ nearly expired food purchase intention.

### Participants

Four Hundred subjects were recruited on Credamo platform in this experiment (61.3% female; *M*_age_=30.8, *SD*_age_=7.93). This experiment uses an inter-group experiment of 2 (time frames: date vs. delay)×2 (food type: healthy vs. unhealthy).

### Procedure

Firstly, the participants were randomly assigned to one of four groups and told that the study was designed to test consumers’ preference for nearly expired food. Similar to experiment 1 and experiment 2, subjects were asked to browse pictures and related information. The stimulus of healthy and unhealthy foods refers to Van Kleef et al. (2018). In the healthy food group, participants were shown whole-grain biscuits, while in the unhealthy food group, they were shown cream biscuits. At the same time, excluding the influence of brand familiarity, this study selected the virtual food brand, LETU. The manipulation of the time frames of the expiration time was the same as experiment 1. The expression of the “date” group was “9th November, 2021”, and the expression of the “delay” group was “in the remaining 45days” (see Supplementary Figure C for specific experimental stimuli). Then, participants answered questions about nearly expired food purchase intention, health perception and time perception (Leboeuf, 2006). The measurement of nearly expired food purchase intention was changed to the scale of (Newman and Howlett, 2016; 1=very unlikely, 9=very likely). The items are: “When you see the product, how likely would you be to buy it?” “When you see the product, how likely would you be to recommend it to others?” (*α*=0.813). The measurement of health perception is: “How healthy is the product”(1=very unhealthy, 9=very likely; Choi et al., 2016). Finally, the subjects filled in demographic information and were told the true purpose.

### Results

#### Manipulation Check

The ANOVA with the intention to purchase nearly expired food as the dependent variable showed that the main effect of time frames was not significant (*F* (1,396)=1.33, *p*=0.249); The main effect of food type is significant (*F* (1,396)=3.94, *p*=0.048); the interaction effect of time frames and food type is not significant (*F* (1, 396)=1.60, *p*=0.206).

#### Time Perception

The analysis results of variance with time perception as the dependent variable show that the main effect of time frames was significant [*F* (1,396)=5.87, *p*=0.016]. The main effect of food type is significant [*F* (1,396)=3.94, *p*=0.048]; the interaction effect of time frames and food type is significant [*F* (1, 396)=7.79, *p*=0.006]. Specifically, for healthy food, compared with the date, the participants in the delay group have higher purchase intention of nearly expired food [*M*_delay_=5.66, *SD*_delay_=2.05, *M*_date_=4.52, *SD*_date_=2.19; *F* (1,396)=13.59, *p*<0.001]. For unhealthy food, there is no significant difference in the time perception of the two conditions [*M*_delay_=4.62, *SD*_delay_=2.29, *M*_date_=4.70, *SD*_date_=2.15; *F* (1,396)=0.07, *p*=0.795].

#### Purchase Intention

**Figure 3:**
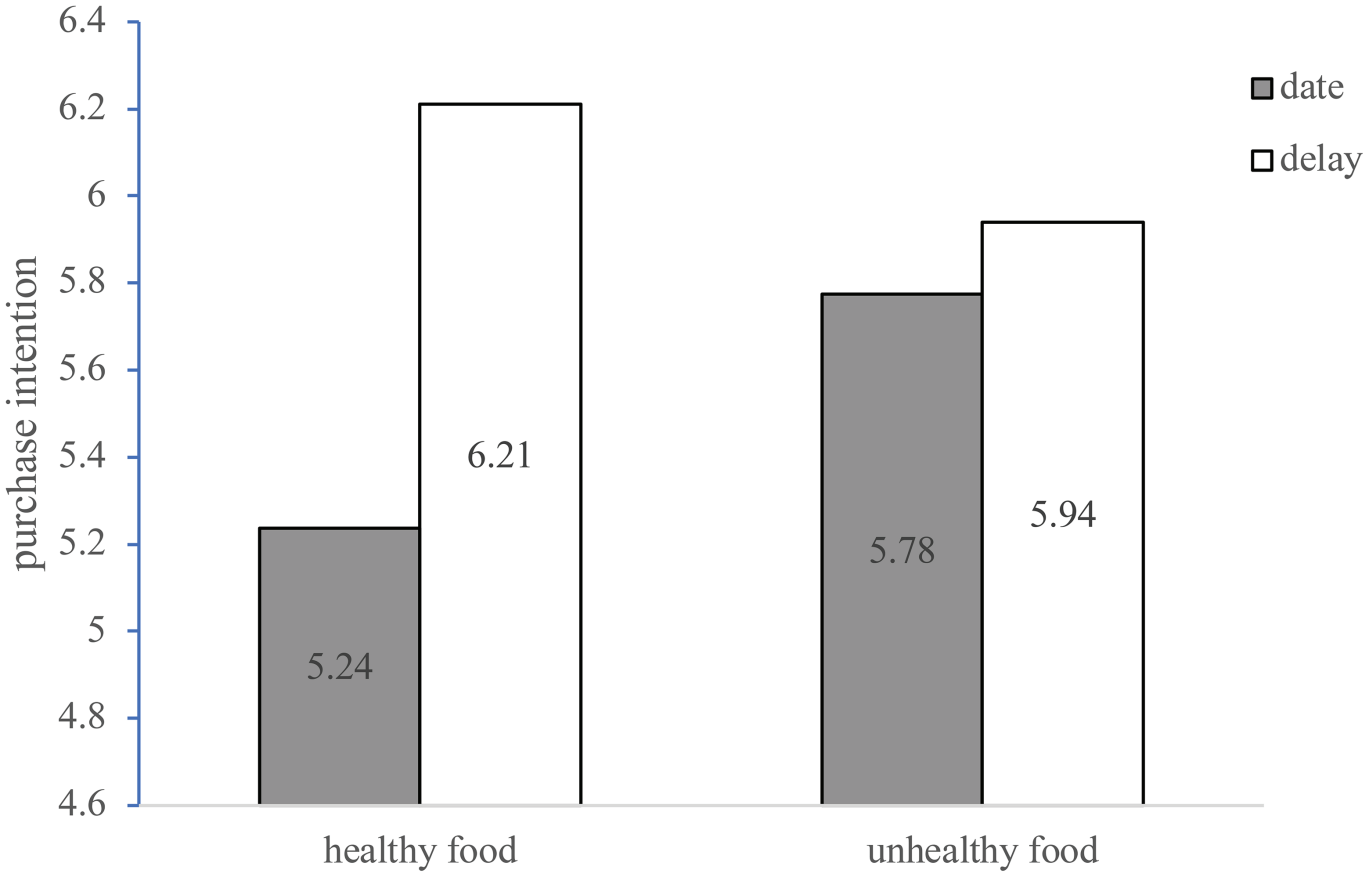
The Moderating Effect of Food Type.

The ANOVA with the intention to buy nearly expired food as the dependent variable showed that the main effect of time frames was significant [*F* (1, 396) =7.78, *p*=0.006]. The main effect of food type is not significant [*F* (1, 396)=0.42, *p*=0.517]; the interaction effect of time frames and food type is significant [*F* (1, 396)=3.92, *p*=0.048]. The result shows a simple effect analysis to the interaction, that for healthy food, compared with date, the participants in the delay have higher purchase intention of nearly expired food [*M*_delay_=6.21, *SD*_delay_=1.89, *M*_date_=5.24, *SD*_date_=2.16; *F* (1,396)=11.37, *p*=0.001]. For unhealthy food, there is no significant difference in the purchase intention of the two conditions [*M*_delay_=5.94, *SD*_delay_=1.99, *M*_date_=5.78, *SD*_date_=2.11; *F* (1,396)=0.33, *p*=0.568]. The results are shown in Figure 3.

#### Model Analysis of Adjusted Mediation Effects

Referring to Hayes’ Model 7(2013), with a sampling of 5,000, In healthy food, the result of the intermediary test is significant (LLCI=0.2559, ULCI=0.8440), indicating that time perception plays a mediating role in the influence of time frames on purchase intention. In the case of unhealthy food, the mediation test results were not significant (LLCI=−0.3199, ULCI=0.2607), indicating that food type did not play a mediating role in the influence of time frames on purchase intention.

### Discussion

The moderating effect of food type was confirmed at Experiment 3, that is, when the food is healthy food, compared with the date, the delay can make consumers perceive the remaining shelf-life longer, and then increase consumers’ purchase intention of nearly expired food. When the food is unhealthy, there was no significant difference in the perception of the remaining shelf-life time in different time frames, so there was no significant difference in the purchase intention of nearly expired food, thus revealing the applicable situation where different time frames had an impact on the purchase intention of nearly expired food.

## General Discussion

### Conclusion

With the rise of field in nearly expired food, how to carry out reasonable publicity on nearly expired food to increase sales volume is the focus for many merchants. Based on the attention gate theory, this study focuses on the influence of different time frames on consumers’ willingness to buy nearly expired food and its effect of internal mechanism. The main conclusions of this study are as follows: (1) compared with the date, consumers are more willing to buy nearly expired food in the delay; (2) Time perception of remaining shelf-life played a mediating role towards the effect of consumer’s buy willingness to nearly expired food under the time frame. In particular, compared with the date, the consumer’s perception of the remaining shelf-life is longer in the delay, so the consumer’s willingness to buy nearly expired food is higher; (3) The food type plays a moderating role in the influence of the time frames on consumers’ willingness to buy nearly expired food. When the food type is healthy, compared with the date, consumers have a longer time perception of the remaining shelf-life when expiration time is framed by delay, thus having higher willingness to buy nearly expired food. When the food type is unhealthy, there is no significant difference between the impact of the time frames on time perception, thus there is no significant difference in consumers’ nearly expired food purchase intentions.

### Theoretical Contribution

This study has a theoretical contribution to the related literature on time frame, purchase intention to nearly expired food, and attention gate theory. Firstly, based on the attention gate theory, this study explored the antecedents of consumers’ purchase intentions to nearly expired food from the time frame, which is a new time dimension, expanded and improved the research framework of theory to nearly expired food. Previous studies on purchase intentions to the nearly expired food mainly focused on pricing (Theotokis et al., 2012; Aschemann-Witzel, 2018), date tags (Collart and Interis, 2018; Wilson et al., 2018; Samotyja and Sielicka-Różyńska, 2021), consumers’ personal characteristics and contexts (de Hooge et al., 2017; Konuk, 2018; do Carmo Stangherlin et al., 2020). Although a few studies have explored the applicable context of the date label of nearly expired food, there were no researchers who have explored the mechanism of the impact of different time frames on consumers’ purchase intention of nearly expired food. Based on the attention gate theory, this research explores the impact of different time frames on consumers’ purchase intention to the nearly expired food, which expands the theoretical framework of pre-influencing factors of nearly expired food purchase intention. At the same time, most of the existing studies have focused on consumers’ aversion behaviors of nearly expired foods and have not proposed effective measures other than offering price discounts to alleviate their aversion to nearly expired foods. This research proposes to change the time frame of expiration time to increase consumers’ perception of remaining shelf-life, thereby reducing the aversion to nearly expired food and increasing the willingness to purchase nearly expired food.

Secondly, this study extends the research on the influence of time frames on consumer purchasing behavior. Previous studies have mainly explored the theoretical mechanism of the date or the delay towards individual intertemporal selection and goal pursuit (Read et al., 2005; Munichor and Leboeuf, 2018), while ignoring the principle of action of influence on the purchase of products with important time characteristics. This study confirms that under the delay, individuals will devote more attention resources to the time length information, the investment of attention resources will give consumers a longer time perception of remaining shelf-life. It further reveals how individual attention affects time estimation and perception, and deepens the causal logic relationship between time information processing and attention. At the same time, the study further confirmed that the remaining shelf-life time perception positively affects consumers’ purchase intention to nearly expired food, thus establishing the theoretical relationship between time frames and purchase intention to nearly expired food, and verifying the mediating role of time perception, which is the deepening and promotion of the relevant research of time perception, and the enrichment and extension of the attention gate theory as well.

In the end, this study introduces the moderating effect of food type. By focusing on the important dimension of food type, this research explores the theoretical relationship between the length of remaining shelf-life and the purchase intention of nearly expired food, and reveals the important role of food type in the influence of time frames on the purchase intention of nearly expired food. The conclusions of the research further illustrate the connection between the type of food at the impending period and consumer decision-making. When the nearly expired food is a healthy food, the consumer’s decision-making is more related to time-dimensional information, and when the nearly expired food is an unhealthy food, consumers’ decision-making pays less attention to time dimension information. Take the food type as an important framework variable in the theoretical research of nearly expired food into the analysis system, extending the research of dimensional characteristics of time variables.

### Management Contribution

The research conclusion has extensive practical value. Food waste is an important social problem, and our research conclusions can promote the sale of nearly expired food and effectively alleviate the problem of food waste. First of all, the conclusion of this research provides some practical guidance for online marketers to reasonably publicize the time information of nearly expired food. Marketers, in online shopping scenarios, will use eye-catching slogans such as “there are still xxx days” or “day/month/year” to promote nearly expired food. However, how the actual effect was not known by the merchants. According to the results, when the expiration time is framed by delay, consumers will have a higher willingness to buy nearly expired food than the date. Therefore, enterprises should use the delay to express the expiration date of food, so as to increase consumers’ purchase behavior to nearly expired food. With the development of intelligent offline product information, electronic tags are increasingly used in stores, and the research conclusions can also be applied to offline consumption scenarios. Secondly, pay attention to how to improve consumers’ time perception. This study confirmed that the perception of remaining shelf-life has a positive effect on the purchase intention of nearly expired food. Therefore, online marketers should pay attention to how to increase consumers’ perception of the remaining shelf-life. In addition to changing the publicity method of food expiration date, merchants can also increase individuals’ time perception by controlling the color of the website environment, the background music of the page and other elements, thus increasing their purchase of nearly expired food. At the same time, it is also possible to transfer consumers’ excessive attention to the expiration date of nearly expired food through the perspective of protecting resources, so that consumers can pay attention to the benefits brought by the purchase of nearly expired food, and then increase the purchase behavior. Finally, adjust propaganda strategy according to different food types. For healthy food, merchants should pay attention to the expression and publicity of its time information, decrease consumers’ perception of risk, and then promote consumers’ purchasing behavior. For unhealthy food, businesses can pay attention to the publicity of taste and other aspects, so as to increase consumers’ purchase intention.

### Limitation and Future Directions

This research still has many deficiencies and needs to be improved. To begin with, expand the research perspective of time dimension. This research pays close attention to the influence of the date and the delay on the willingness to buy nearly expired food. Existing studies have found that the time precision (number of months or days; Chandran and Menon, 2004; Charles Zhang and Schwarz, 2012) and time unit (default or uncommon units; Lembregts and Pandelaere, 2013) have an influence on consumer psychology. Future research can expand the research perspective of the time dimension and explore its influence mechanism on nearly expired food from time precision and unit, enriching research on the time dimension of nearly expired food. Secondly, we further explore the role of other regulatory variables. This study introduces and confirms the moderating effect of food type, but there still exist other variables to be tested and which have practical value in the context of nearly expired food consumption. From the perspective of consumers, the role of individual character factors in consumer purchase behavior can further be explored in the future. Finally, increase the research methods. This study only uses an experimental method to test the hypothesis, but it does not further explore the effect of the research conclusions in practice. Second-hand data online-offline and field experiments can be added in future research.

## Data Availability Statement

The raw data supporting the conclusions of this article will be made available by the authors, without undue reservation.

## Funding

This research was supported by the Fundamental Research Funds for the Central Universities, and the Research Funds of Renmin University of China (20XNL013).

## Author Contributions

MS is responsible for the construction and later revision of this paper. YZ is the co-first author and responsible for the later revision of this paper. XL is the correspondence and responsible for theoretical literature review, article writing and adjusting. LM is the co-correspondence and responsible for the adjusting. All authors contributed to the article and approved the submitted version.

## Conflict of Interest

The authors declare that the research was conducted in the absence of any commercial or financial relationships that could be construed as a potential conflict of interest.

## Publisher’s Note

All claims expressed in this article are solely those of the authors and do not necessarily represent those of their affiliated organizations, or those of the publisher, the editors and the reviewers. Any product that may be evaluated in this article, or claim that may be made by its manufacturer, is not guaranteed or endorsed by the publisher.

## Footnotes

^1^Taobao, Jingdong and Suning Tesco are well-known online shopping retail platforms in China. Taobao: https://www.taobao.com/ Jingdong: https://www.jd.com/ Suning Tesco: https://www.suning.com/

^2^“iiMedia Report |2020 China’s temporary food industry Market Analysis and Consumer Research Report.” https://www.iimedia.cn/c400/76576.html

^3^HAO SHIQI is the e-commerce platform for selling nearly expired food. HAO SHIQI: https://haoshiqi.net/

^4^Beijing Administration for Industry and Commerce. “Zone System of Nearly Expired Food Sales.” http://www.gov.cn/jrzg/2012-02/03/content_2057892.htm
