# Supplementary figures and images for: The Influence Exerted by Time Frames on Consumers’ Willingness to Buy Nearly Expired Food
Source: Front Psychol. 2021 Dec 20;12:790727. doi: 10.3389/fpsyg.2021.790727 (PMC8722527; doi:10.3389/fpsyg.2021.790727)

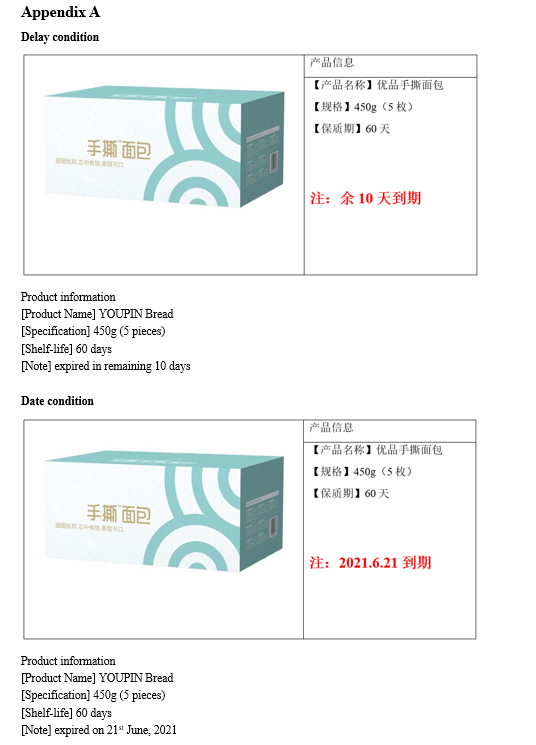

Supplement: Supplementary file 1 [file Image_1.png]

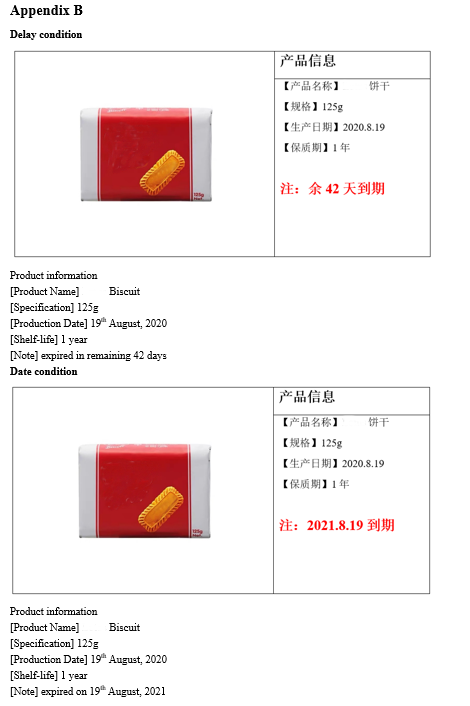

Supplement: Supplementary file 2 [file Image_2.png]

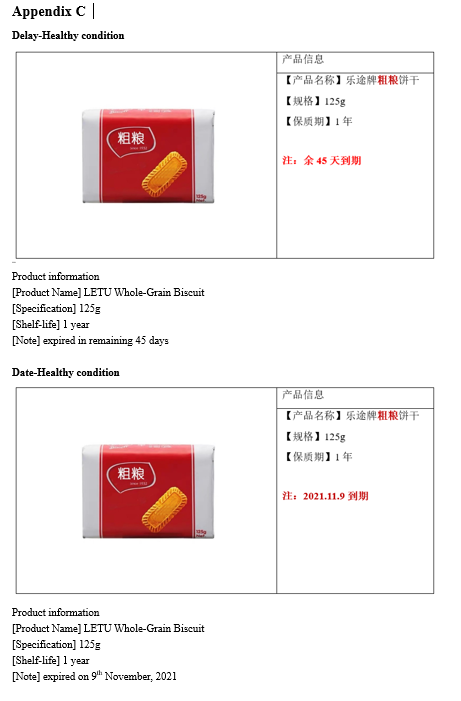

Supplement: Supplementary file 3 [file Image_3.png]

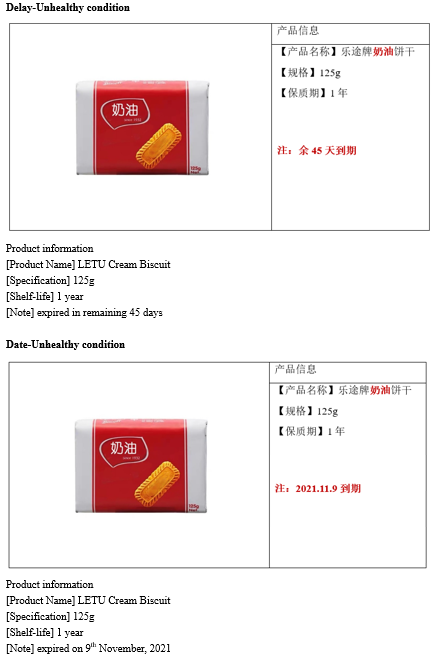

Supplement: Supplementary file 4 [file Image_4.png]
